# Efficient tissue-type specific expression of target genes in a tetracycline-controlled manner from the ubiquitously active *Eef1a1* locus

**DOI:** 10.1038/s41598-019-57052-z

**Published:** 2020-01-14

**Authors:** Kazuhito Sakamoto, Patrick D. Rädler, Barbara L. Wehde, Aleata A. Triplett, Hridaya Shrestha, Rosa-Maria Ferraiuolo, Foued Amari, Vincenzo Coppola, Apostolos Klinakis, Argiris Efstratiadis, Kay-Uwe Wagner

**Affiliations:** 1 Eppley Institute for Research in Cancer and Allied Diseases, University of Nebraska Medical Center, 985950 Nebraska Medical Center, Omaha, NE 68198-5950 USA; 2 Department of Oncology, Wayne State University School of Medicine and Tumor Biology Program, Barbara Ann Karmanos Cancer Institute, 4100 John R, EL01TM, Detroit, MI 48201 USA; 3 Genetically Engineered Mouse Modeling Core, Comprehensive Cancer Center, Ohio State University, Columbus, Ohio USA; 4 Department of Cancer Biology and Genetics, College of Medicine and Comprehensive Cancer Center, Ohio State University, Columbus, Ohio USA; 5 Center of Basic Research, Biomedical Research Foundation of the Academy of Athens, 11527 Athens, Greece

**Keywords:** Mouse, Genetic engineering, Cancer models

## Abstract

Using an efficient gene targeting approach, we developed a novel mouse line that expresses the tetracycline-controlled transactivator (tTA) from the constitutively active *Eef1a1* locus in a Cre recombinase-inducible manner. The temporally and spatially controlled expression of the EF1-LSL-tTA knockin and activation of tTA-driven responder transgenes was tested using four transgenic lines that express Cre under tissue-specific promoters of the pancreas, mammary gland and other secretory tissues, as well as an interferon-inducible promoter. In all models, the endogenous *Eef1a1* promoter facilitated a cell-type-specific activation of target genes at high levels without exogenous enhancer elements. The applicability of the EF1-LSL-tTA strain for biological experiments was tested in two studies related to mammary gland development and tumorigenesis. First, we validated the crucial role of active STAT5 as a survival factor for functionally differentiated epithelial cells by expressing a hyperactive STAT5 mutant in the mammary gland during postlactational remodeling. In a second experiment, we assessed the ability of the EF1-tTA to initiate tumor formation through upregulation of mutant KRAS. The collective results show that the EF1-LSL-tTA knockin line is a versatile genetic tool that can be applied to constitutively express transgenes in specific cell types to examine their biological functions at defined developmental stages.

## Introduction

Over the past 20 years, tetracycline (tet)-controlled expression systems have been invaluable tools to study the biological effects of a reversible overexpression of transgenes in genetically engineered mouse models. Research teams have generated a collection of mouse strains that allow for temporal and spatial regulation of genes *in vivo* by targeting the tet-controlled transactivator (tTA) protein to certain cell types using tissue-specific promoters. The ability of the tTA to activate tet-operator/promoter (TetO)-driven effector transgenes is controlled by administration of doxycycline (Dox), which is an effective and commonly used tetracycline derivative. Most genetic mouse models that are being applied to control the expression of transgenes in a ligand-controlled manner utilize one of the two main types of the tet-controlled expression systems that were pioneered by Gossen and Bujard in the early and mid-1990s. Specifically, the Tet-OFF system employs a Dox-responsive transactivator protein (tTA) that is being rendered transcriptionally inactive in the presence of the antibiotic^1^. In contrast, the Tet-ON system was devised on a reverse transactivator (rtTA) consisting of a mutated tetracycline repressor domain that becomes active in the presence of the ligand^2^.

Soon after Furth and coworkers^3^ established the binary tet-controlled expression system as a genetic tool in mice, they developed tTA transgenic lines under regulation of the long terminal repeat (LTR) of the mouse mammary tumor virus (MMTV) to target the expression of oncogenes to the mammary gland and other secretory tissues^4^. They were also first to apply this model to study divergent roles of the SV40 large T (TAg) oncoprotein in cancer initiation and progression^5^. The subsequent development of MMTV-rtTA transgenics by Gunther *et al*. permitted a more efficient activation of oncogenes such as ErbB2 in the mammary gland^6,7^. To target transgenes exclusively to the mammary gland in a ligand-inducible manner, our team placed the rtTA under control of the endogenous *whey acidic protein* (*Wap*) gene^8^, and we also developed new MMTV-tTA lines that permit a targeted expression of genes to the epithelium of the prenatal and postnatal mammary gland^9^. Without the need for a continuous administration of Dox, the tTA is useful for long-term expression studies of weak oncogenic drivers. Despite superior applicability of all these transactivator strains, they still have several limitations. Except for the Wap-rtTA knockin, MMTV-based lines express the transactivator protein in secretory tissues other than the mammary gland such as the salivary gland and seminal vesicle. Moreover, the degree of transgene activation within epithelial lineages of the mammary gland varies greatly depending on the hormonal milieu during the gestation cycle, and the ligand-controlled expression of target genes cannot be restricted to individual epithelial subtypes within the developing mammary gland.

An experimental approach to overcome most of these limitations is to place the expression of the transactivator under the control of a ubiquitously active promoter or endogenous housekeeping gene. The cell-type-specific activation of the tTA is being accomplished by expression of Cre recombinase that will remove a transcriptional *Stop* sequence located between the promoter and the coding sequence of the tTA/rtTA. We previously tested a promising knockin line where the rtTA is driven from the endogenous *Rosa26* locus^10^ in combination with the MMTV-Cre or WAP-Cre transgenes and various responder genes such as TetO-Stat5 and TetO-CyclinD1^11,12^. Despite extensive Cre recombination in selected tissues such as the skin in the MMTV-Cre-based model, none of the females exhibited an expression of the TetO-driven target genes in the mammary gland at levels that were comparable to those observed in crosses with the Wap-rtTA or MMTV-tTA lines (unpublished). In another attempt, we generated embryonic stem (ES) cell-based transgenic lines that express the tTA under the CMV-enhanced chicken β-actin (CAG) promoter in a Cre-inducible manner^13^. Although the CAG-tTA was uniformly active in ES cells and in preimplantation embryos, we found that the transgene was progressively silenced in selected tissues of prenatal and adult mice, including the mammary and salivary glands, prostate, and most hematopoietic cells. High expression of the CAG-tTA was only retained in the brain, skin, muscle, heart, as well as the exocrine and endocrine pancreas.

Building on our collective experiences, we have now completed the development of a novel mouse line that expresses the tTA from the ubiquitously active, endogenous *Eef1a1* (EF1, EF1α) locus. In addition to validating the correct tissue-specific activation of the EF1-LSL-tTA and Dox-controlled expression of TetO-driven reporters in four transgenic lines expressing Cre recombinase, we also performed two experiments to demonstrate the applicability of the EF1-LSL-tTA strain for biological studies. The collective observations suggest that the EF1-LSL-tTA knockin can be applied to a great variety of experimental approaches that examine the role of genes in particular cell types at defined stages of development. The superiority of the constitutively active *Eef1a1* locus to drive the expression of oncogenes and to examine their biological significance in diverse cellular subtypes, including their progressively transforming descendants that undergo de- or trans-differentiation, is discussed.

## Results

### Superior targeting efficiency using a promoter-less neomycin selection marker to create the EF1-LSL-tTA knockin strain

**Figure 1:**
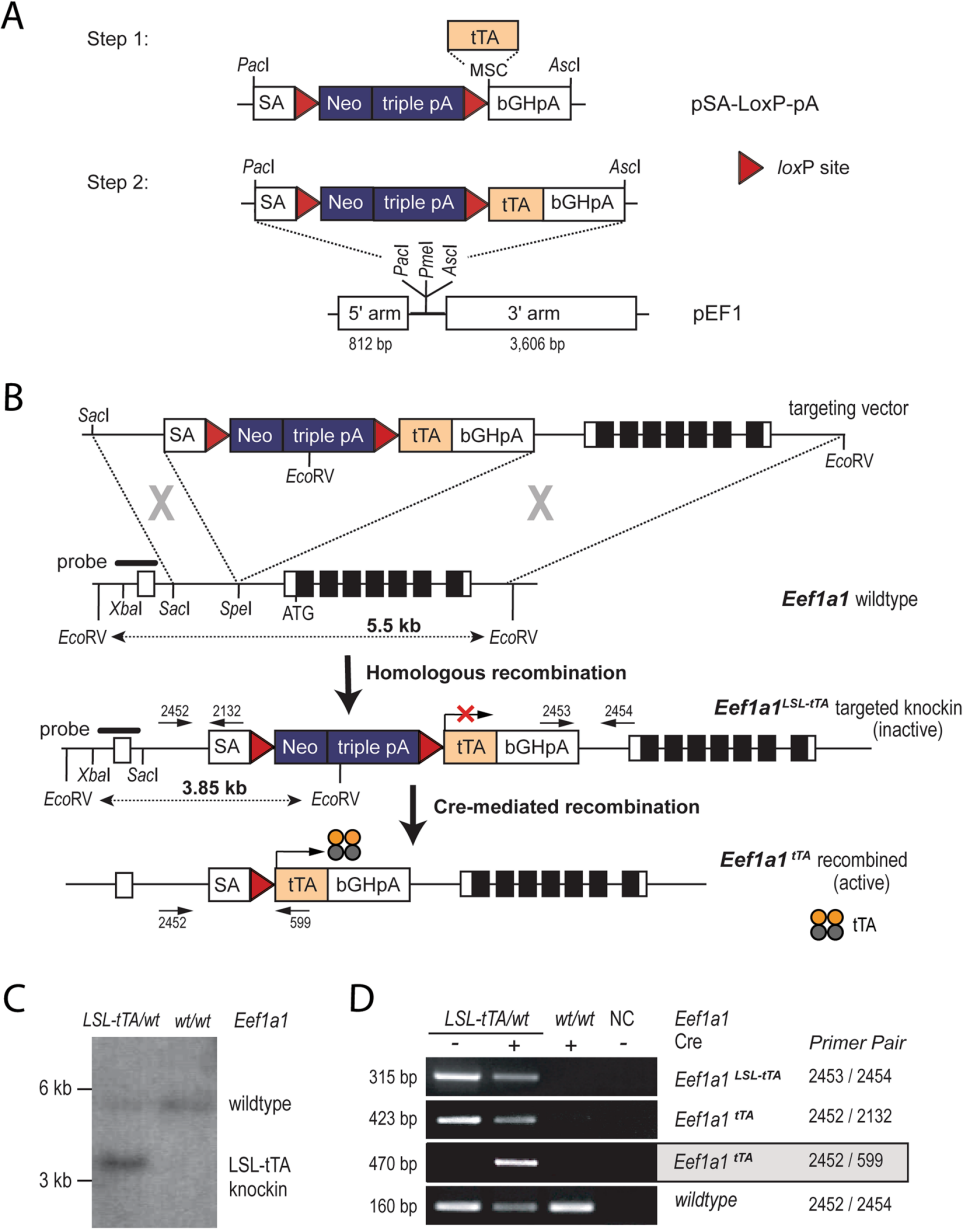
Targeted insertion of the LSL-tTA into the endogenous *Eef1a1* (EF1) locus. (**A**) Two-step cloning approach of the EF1-LSL-tTA gene targeting vector. (**B**) Strategy for the targeted knockin of the LSL-tTA upstream of the first coding exon using homologous recombination. (**C**) Southern blot analysis using an *Eco*RV restriction digest in combination with a 3′ external probe to verify the correct targeted insertion of the LSL-tTA. (**D**) PCR genotyping on genomic DNA of fibroblasts from an EF1-LSL-tTA (*Eef1a1*^*LSL-tTA*/+^) mouse with and without Cre recombinase as well as a wildtype control (*Eef1a1*^+/+^). Abbreviations: L, *lox*P site; S, transcriptional *Stop* sequence; tTA, tetracycline-controlled transactivator; SA, splice acceptor site; Neo, neomycin selection marker; pA, polyadenylation signal; bGH, bovine growth hormone; ATG, start codon; MSC, multi-cloning site; arrows indicate the location of primer sequences.

To generate EF1-LSL-tTA knockin mice, a conventional homologous recombination approach in mouse embryonic stem (ES) cells was used for the targeted insertion of the tetracycline-controlled transactivator (tTA) into the endogenous *eukaryotic translation elongation factor 1 alpha 1* (*Eef1a1*) locus. The tTA is preceded by a transcriptional *Stop* sequence (i.e., three polyA signals), which is surrounded by two *loxP* sites (LSL). The construction of the targeting vector is shown in Fig. 1A and described in detail in the *Material and Methods* section. Unique features of the targeting vector are the use of a promoter-less neomycin selection marker and the relatively short homology sequence on the 3′ end of the construct that allows for an efficient screening procedure of ES cell clones by PCR with primers that bind inside and outside of the *Eef1a1* targeted region. A splice acceptor site (SA) mediates the expression of the neomycin selection marker from the ubiquitously expressed *Eef1a1* locus in targeted ES cell clones without the need for an additional 3′ thymidine kinase (TK)-expressing cassette and ganciclovir-based selection against random integration events (Fig. 1B). The SA was strategically placed in front of the LSL to facilitate a constitutive expression of the tTA in place of neomycin after the Cre-mediated excision of the selection marker and *Stop* cassette.

Following electroporation of the linearized targeting vector into S1B6a ES cells and Geneticin selection, 36 clones were expanded and pre-screened by PCR for the targeted insertion. Twenty-seven targeted clones (75%) were identified by PCR, and we confirmed the correct targeted insertion of the EF1-LSL-tTA construct by *Eco*RV restriction digest and Southern blot using a 5′ external probe that was not part of the targeting vector (Fig. 1B). Similar to the Southern blot shown in Fig. 1C, all eight clones exhibited the expected 3.85 kb band for the targeted EF1-LSL-tTA locus in addition to the 5.5 kb band for the wildtype *Eef1a1* allele. Although we did not assess the targeted insertion in all PCR negative clones by restriction digest, two of them that were included in the Southern blot screen clearly showed the 3.85 kb targeted band, suggesting that the actual targeting frequency may have exceeded 75 to 80%. It is important to note that the successful targeting of the *Eef1a1* locus at such a high frequency with the help of this strategy was only accomplished when pure 129/Sv or hybrid ES cells were used, i.e., those lines that were isogenic to the source DNA for the construction of the homology arms of the targeting vector. Four attempts to insert the EF1-LSL-tTA and two other constructs into the *Eef1a1* locus of C57BL/6-derived ES cells were unsuccessful.

Correctly targeted ES cell clones were injected into C57Bl/6J albino blastocyst and transferred to ICR pseudopregnant females. The injection yielded numerous offspring with 40 to 90% chimerism. Chimeric males were crossed with C57Black Swiss to transmit the targeted EF1-LSL-tTA allele through the germline. In addition to Southern blot, the presence of the 3′ and 5′ end of the inserted transgene was confirmed by PCR (Fig. 1B,D; primer pairs 2453/2454 and 2452/2132). The ability of Cre recombinase to correctly excise the transcriptional *Stop* sequence (Fig. 1B, bottom) was tested by expressing Cre in fibroblasts derived from EF1-LSL-tTA transgenics and controls. The excision of the *Stop* sequence was confirmed by PCR (Fig. 1D, primer pair 2452/599). The insertion of the SA and LSL-tTA in front of the first coding exon resulted in a functional *Eef1a1* null allele. In support of the multifaceted molecular roles of EeF1A1, it was not surprising that we never obtained any homozygous EF1-LSL-tTA knockin mice regardless of the genetic background. Consequently, the EF1-LSL-tTA knockin allele is being maintained in a heterozygous state. Heterozygous mutants of both genders are fertile and do not show any phenotypic abnormalities, even after the targeted allele was backcrossed for more than ten generations into pure FVB/N or C57BL/6 backgrounds.

### Pdx1-Cre directs the EF1-mediated expression of the tTA predominantly, but not exclusively, to the pancreas

**Figure 2:**
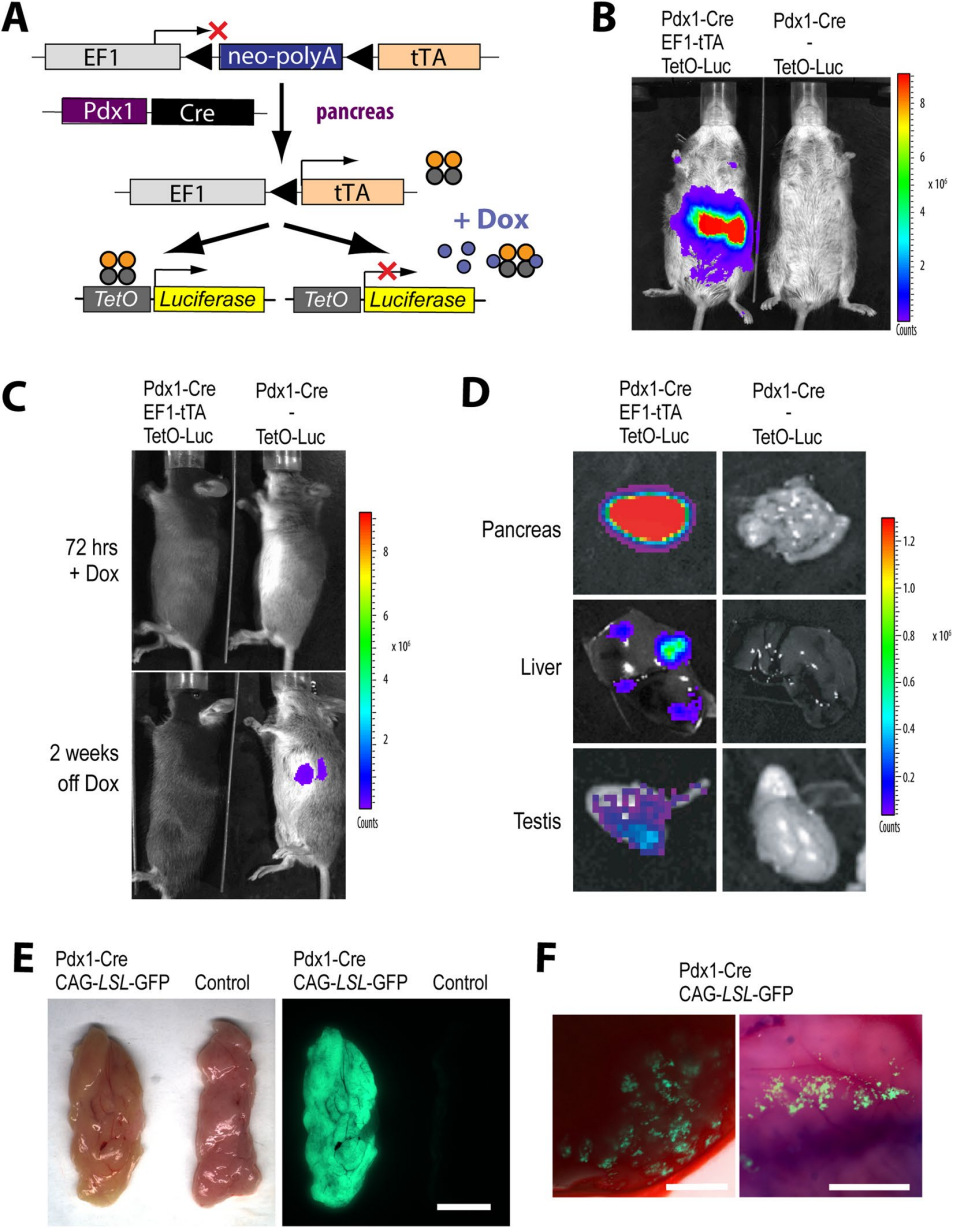
Pdx1-Cre-directed, constitutively high expression of the tTA and its responder genes to the pancreas. (**A**) Diagram of a genetically engineered mouse model carrying 3 transgenes (Pdx1-Cre, EF1-LSL-tTA, and TetO-Luciferase) for a temporally and spatially controlled expression of genes in the pancreas. (**B**,**C**) *In vivo* bioluminescence imaging of a Pdx1-Cre EF1-LSL-tTA TetO-Luc transgenic mouse and a littermate control without the EF1-LSL-tTA knockin allele. (**D**) Luciferase expression in pancreas, liver, and testis. (**E**,**F**) Brightfield and GFP images of unfixed pancreas (**E**) and liver (**F**) tissues from Pdx1-Cre/CAG-LSL-GFP double transgenic mice; bars represent 5 mm (**E**) and 50 µm (**F**).

To test the tissue-specific activation of the EF1-LSL-tTA, we generated triple transgenic mice that carry the Pdx1-Cre and TetO-Luciferase reporter transgenes in addition to the EF1-LSL-tTA knockin allele (Fig. 2A). As determined by *in vivo* bioluminescence imaging, a strong tTA-mediated expression of luciferase was confined to the abdominal region of triple transgenic animals, and we did not detect any background activation of the sensitive reporter transgene in the absence of the EF1-LSL-tTA (Fig. 2B). Administration of Dox for 48 to 72 hours was sufficient to completely suppress the EF1-tTA-mediated expression of luciferase, and the activation of the reporter transgene recovered within two weeks following the removal of the antibiotic from the drinking water and its clearance from the tissues (Fig. 2C). A necropsy following bioimaging and reexamination of the bioluminescence signal within individual organs revealed that a very strong EF1-tTA-mediated expression of luciferase was present in the pancreas, but a moderate activity of the reporter was also detected in focal areas of the liver and testis. A careful examination of tissues from Pdx1-Cre mice that carry a CAG-LSL-GFP reporter under a fluorescent stereoscope showed that the Cre transgene was predominantly expressed in the pancreas, but it was also active in selected regions of the liver (Fig. 2E,F). The sporadic activation of the Pdx1-Cre in the liver should be considered if this transgene is being used to upregulate oncogenes like mutant KRAS in combination with Cre/lox-based cell lineage tracing to study the potential dissemination of preneoplastic or fully transformed pancreatic cells to the liver as reported earlier^14^.

### Widespread EF1-tTA-driven expression of transgenes in secretory tissues, splenocytes, and skin

**Figure 3:**
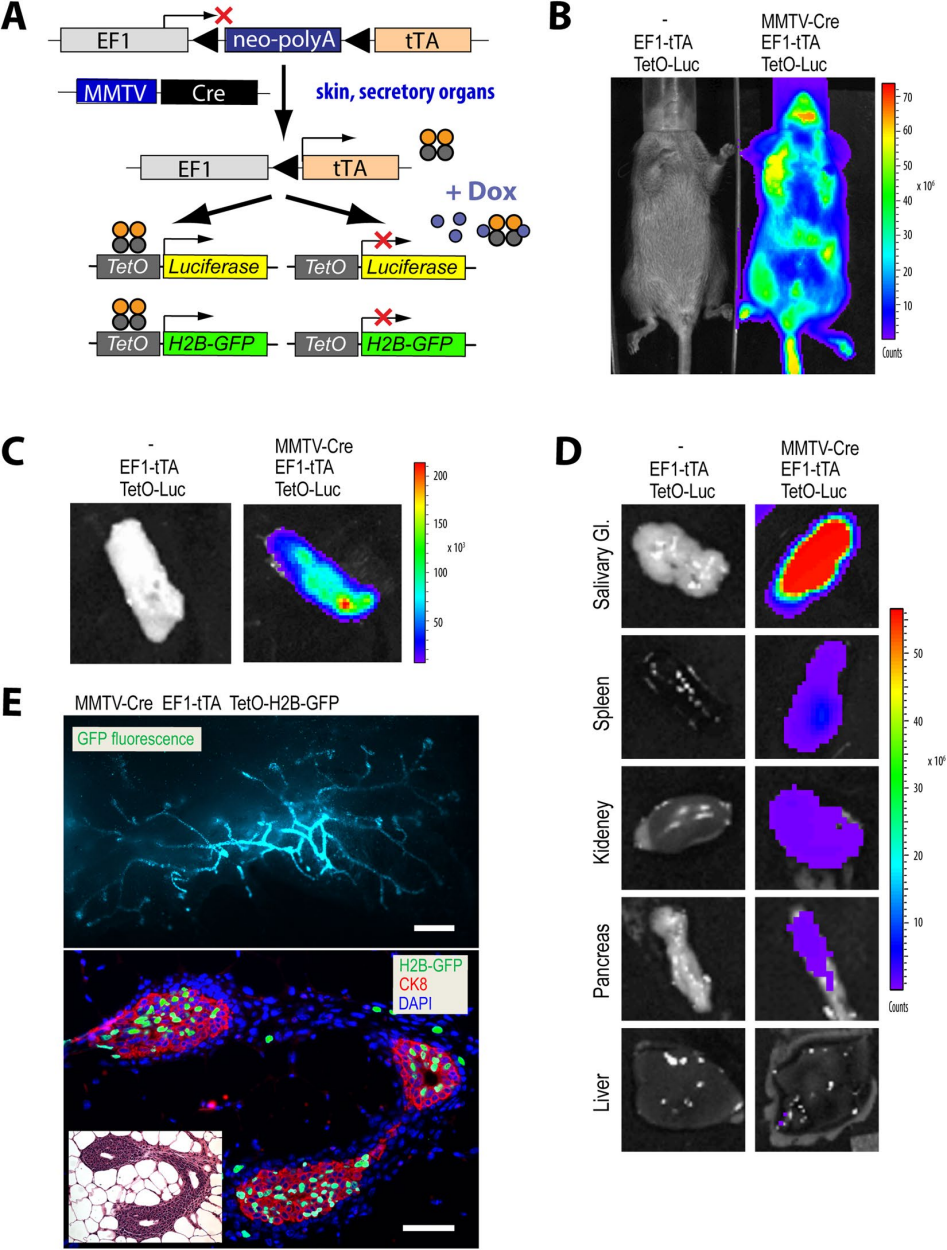
Strong activation of the EF1-tTA in the mammary and salivary glands, skin, and spleen in MMTV-Cre transgenics. (**A**) Schematic of the MMTV-Cre-mediated activation of the EF1-LSL-tTA and TetO-driven reporter genes in secretory tissues and skin. (**B**) *In vivo* bioluminescence imaging of a MMTV-Cre EF1-LSL-tTA TetO-Luc triple transgenic mouse and a littermate control without the MMTV-Cre transgene. Note that the widespread activation of the EF1-tTA in the skin obscures the expression of the TetO-Luc in internal MMTV-Cre target organs. (**C**,**D**) Strong luciferase expression in the #4 inguinal mammary (**C**) and salivary glands (**D**), and moderately high activation in spleen, kidney, and parts of the pancreas. (**D**,**E**) GFP fluorescent image of an unfixed #4 inguinal mammary gland of a virgin MMTV-Cre EF1-LSL-tTA TetO-H2B-GFP female (upper panel; bar, 1 mm); immunofluorescent staining of cytokeratin 8 (CK8) and nuclear GFP (lower panel; bar, 50 µm); DAPI was used as counterstain; inset, hematoxylin/eosin-stained serial section of the same specimen.

Our primary motivation for the development of the EF1-LSL-tTA knockin strain was to constitutively activate transgenes in the mammary gland in a ligand-controlled manner. To test whether the endogenous *Eef1a1* locus is capable of driving a high expression of the tTA to this organ, we generated triple transgenic females that carry the EF1-LSL-tTA knockin in addition to the MMTV-Cre (line D) and the TetO-Luciferase or TetO-H2B-GFP reporters (Fig. 3A). The MMTV-Cre lines that we previously developed have been widely used to delete genes in the mammary gland, but the transgene is also active in other secretory tissues and the skin^15^. As anticipated, MMTV-Cre EF1-LSL-tTA TetO-Luciferase triple transgenic females exhibited a very strong and uniform expression of the reporter in the skin (Fig. 3B). We did not detect any bioluminescent signal in EF1-LSL-tTA TetO-Luciferase double transgenic control mice that do not carry a Cre transgene. To assess the EF1-tTA-mediated expression in the mammary gland and other organs, we performed a necropsy on luciferase-positive triple transgenic females and their controls and repeated the bioluminescence imaging on isolated organs. Following MMTV-Cre-mediated recombination, the EF1-tTA knockin induced a very high expression of the TetO-Luciferase responder gene in the mammary (Fig. 3C) and salivary glands (Fig. 3D). Weaker bioluminescent signals were detected in the spleen, kidney, and pancreas (Fig. 3D).

To determine the cell-type-specific activation of the EF1-tTA, we examined the nuclear expression of a histone-tagged GFP reporter in the mammary gland of nulliparous (virgin) MMTV-Cre EF1-LSL-tTA TetO-H2B-GFP triple transgenic females. When being examined under a fluorescent stereoscope, all mammary gland tissues exhibited a strong expression of the GFP reporter throughout the epithelial ductal tree (Fig. 3E). The majority of GFP-positive nuclei were detected in the luminal epithelium as determined by immunostaining of histological sections against cytokeratin 8 (CK8) and the green fluorescent protein (Fig. 3E).

**Figure 4:**
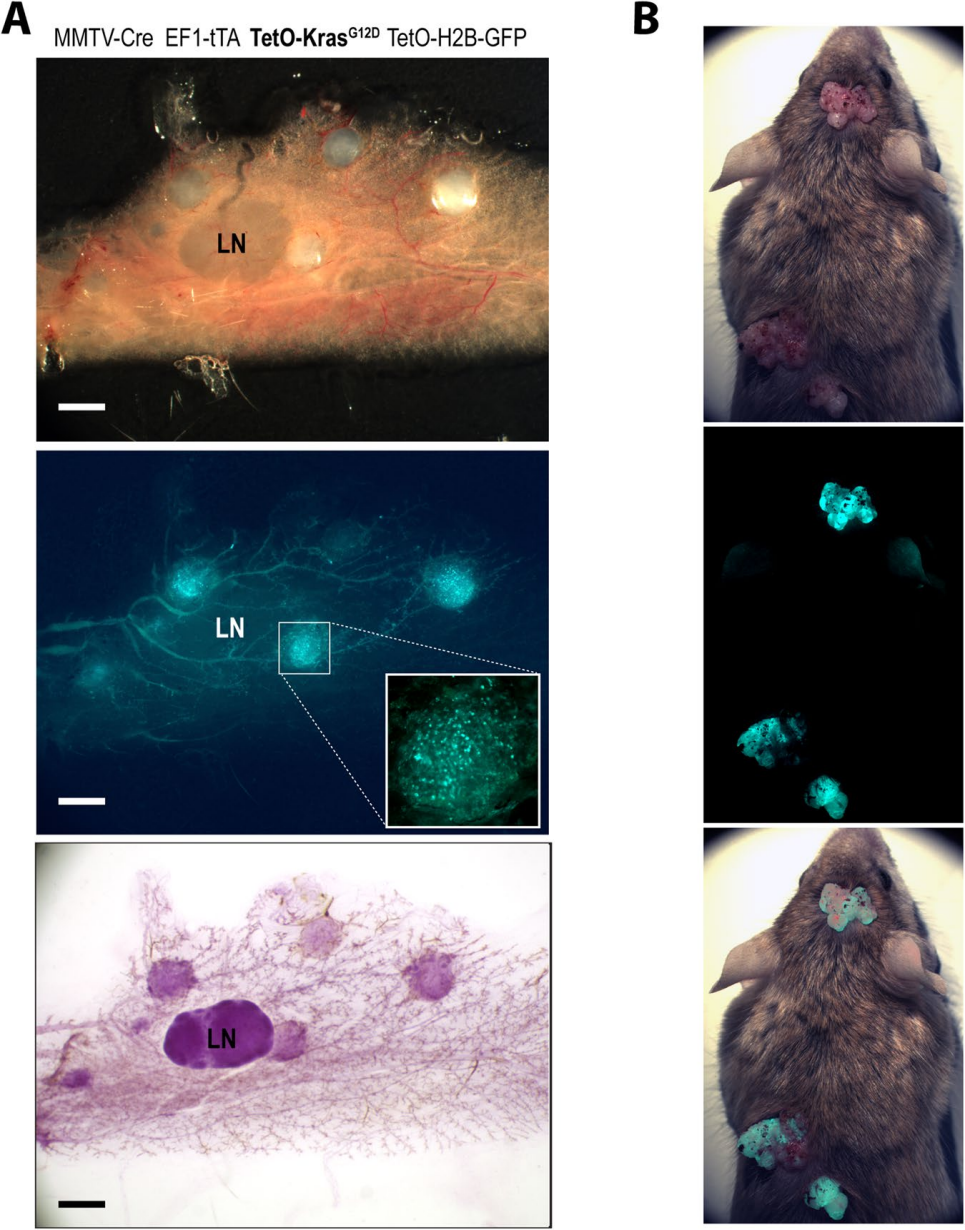
Constitutive expression of oncogenic KRAS under the control of the EF1-tTA in the mammary gland and other MMTV-Cre target tissues. (**A**) Brightfield and GFP fluorescent images (upper, middle panels) of an unfixed #4 inguinal mammary gland of MMTV-Cre EF1-LSL-tTA TetO-KRAS^G12D^ TetO-H2B-GFP quadruple transgenic female; Carmine alum-stained wholemount of the same tissue (lower panel); LN, lymph node; bars, 1 mm. (**B**) Brightfield (upper), GFP (middle), and overlay (bottom) images of a quadruple transgenic mouse that developed multiple skin tumors.

Next, we generated quadruple transgenic females that carry the TetO-Kras^G12D^ transgene in addition to the MMTV-Cre, EF1-LSL-tTA, and the TetO-H2B-GFP reporter to perform an inaugural study that assesses whether the MMTV-Cre-mediated constitutive activation of the EF1-tTA is capable of driving the expression of an oncogene that can initiate the neoplastic transformation of the mammary epithelium. As shown in Fig. 4A, the expression of mutant KRAS led to a multifocal onset of mammary tumors that co-expressed the nuclear GFP reporter. Due to the occurrence of KRAS-induced tumors in other organs, in particular, the skin (Fig. 4B) but also the salivary gland and ovary (not shown), a further examination of mammary cancer progression and metastasis was not conducted in this model. The initial results from this pilot study on tumor initiation as well as the phenotypic consequences of STAT5 overexpression in the mammary gland that are described later demonstrate the value of the EF1-LSL-tTA strain for biological studies.

### Strong activation of EF1-tTA-driven transgenes specifically in secretory mammary epithelial cells and their descendants that survive postlactational remodeling

**Figure 5:**
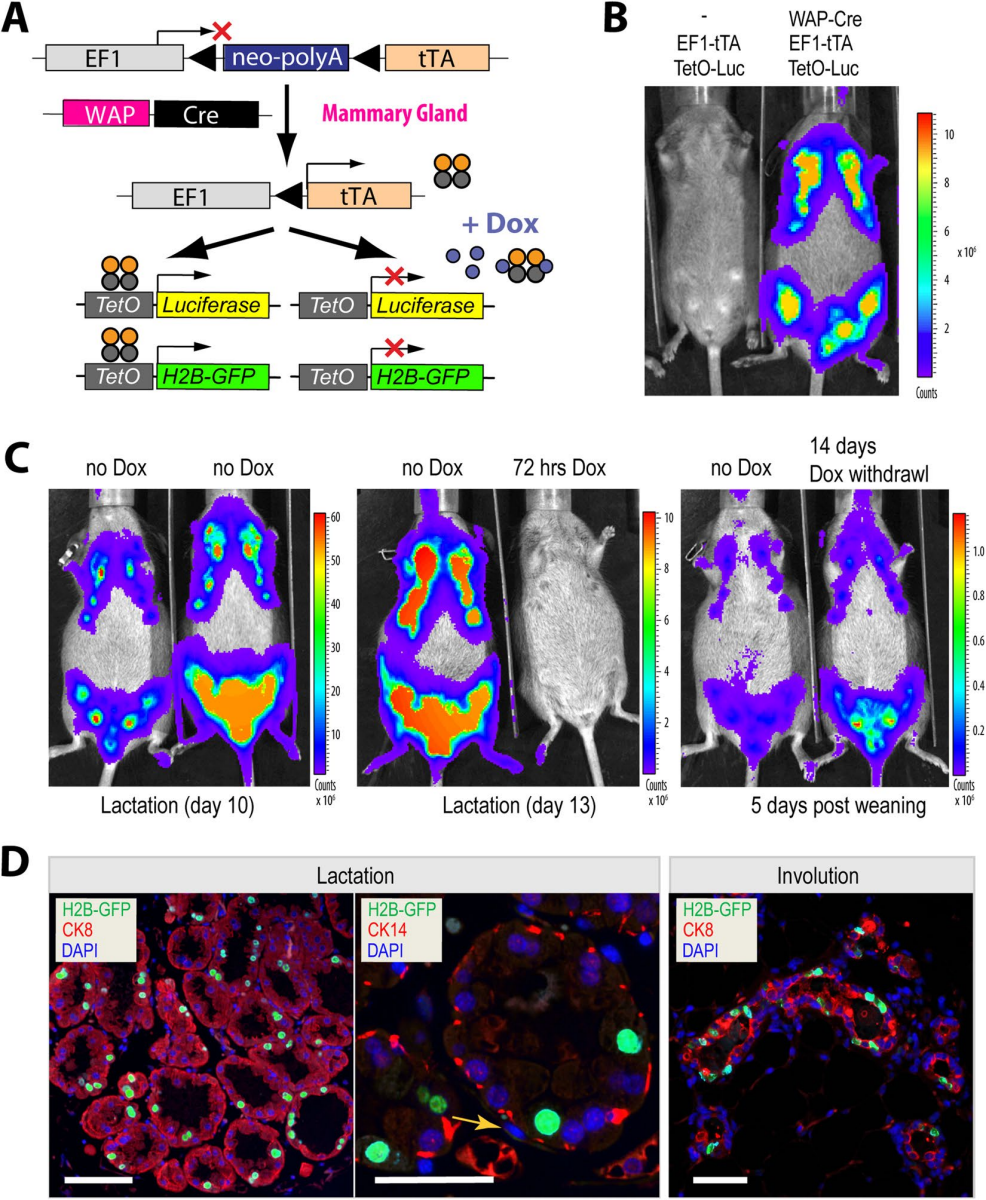
Temporally and spatially controlled expression of transgenes in the developing mammary gland following the WAP-Cre-mediated, constitutive activation of the EF1-tTA. (**A**) Diagram of the WAP-Cre-based mouse model that facilitates a constitutive activation of the EF1-LSL-tTA and its responder genes exclusively in the mammary gland epithelium during and following the first gestation cycle. (**B**) *In vivo* bioluminescence imaging of a WAP-Cre EF1-LSL-tTA TetO-Luc triple transgenic female and a littermate control without the WAP-Cre on day 10 of lactation. (**C**) *In vivo* imaging of two lactating triple transgenic females before (left) and during doxycycline (Dox)-mediated suppression of luciferase activation (middle) and two weeks following Dox-withdrawal (right), which led to a progressive reactivation of the TetO-Luc transgene and its continuous expression during postlactational involution. (**D**) Immunofluorescent staining of nuclear GFP and cytokeratin 8 and 14 (CK8 and CK14) on histological sections of mammary gland tissues during lactation (left, middle) and two weeks after postlactational remodeling (right), DAPI was used as counterstain; bars, 50 µm; yellow arrow points to a typical spindle-shaped nucleus of a myoepithelial cell.

WAP-rtTA knockin mice express TetO-driven responder transgenes in a Dox-inducible manner explicitly in alveolar cells of the mammary epithelium that undergo secretory differentiation during late pregnancy and lactation^8^. Nonetheless, a high level of transgene expression cannot be sustained in these mice because the *whey acidic protein* (*Wap*) gene is being swiftly silenced following the weaning of the young, and most secretory epithelial cells die within a week during postlactational remodeling. To assess whether the tTA under control of the *Eef1a1* locus is generally suitable to direct the expression of transgenes to particular epithelial subtypes within a defined developmental window, we crossed EF1-LSL-tTA knockin mice that carried luciferase or GFP reporter transgenes with WAP-Cre mice (Fig. 5A). We have previously demonstrated that an efficient WAP-Cre-mediated recombination requires at least one full-term gestation cycle and its expression is largely limited to functionally differentiated alveolar cells^16^. Consequently, bioluminescent signals were not detected in the glands of nulliparous WAP-Cre EF1-LSL-tTA TetO-Luciferase triple transgenic females (not shown), but the activation of WAP-Cre caused a robust and strictly mammary-gland specific expression of luciferase during the first pregnancy and lactation period (Fig. 5B). The high EF1-tTA-mediated expression of the reporter could be completely repressed with Dox-medicated drinking water within 48 to 72 hours (Fig. 5C, left and center). The withdrawal of Dox from lactating females led to a reactivation of the TetO-Luciferase transgene within a week to 14 days, and bioluminescent signals were still abundant following the weaning of the offspring when the mammary gland undergoes extensive postlactational remodeling (Fig. 5C, right).

The microscopic analysis of immunostained histological sections of lactating mammary glands from WAP-Cre EF1-LSL-tTA TetO-H2B-GFP triple transgenic females confirmed that GFP-labeled nuclei were present in CK8-positive secretory alveolar cells. Not all alveolar cells exhibited GFP-positive nuclei, which is likely a consequence of the combined mosaic expression patterns of the WAP-Cre and TetO-H2B-GFP transgenes. We did not detect any green fluorescence within the spindle-shaped nuclei of the surrounding CK14-labeled myoepithelial cells (Fig. 5D, left). Using the WAP-Cre mice as a tool to genetically label secretory alveolar cells, we previously made the surprising finding that many functionally differentiated cells survive the postlactational involution process and serve as alveolar progenitors for the expanding lobuloalveolar compartment during subsequent pregnancies^16^. Since many of these genetically labeled cells remained present over the lifespan of parous females following their first gestation cycle, we named these cells parity-induced mammary epithelial cells (PI-MECs)^17^. The histological examination of completely involuted mammary glands of parous triple transgenic females showed that the EF1-tTA-mediated, constitutive expression of the GFP reporter was still present after four or more weeks following the weaning the offspring (Fig. 5D, right). Since PI-MECs do not belong to a long-term label-retaining epithelial subtype^8^, the location of the GFP-positive cells within virtually all terminal ducts indicate that the tTA expression under the *Eef1a1* locus remained constitutively active within PI-MECs. In support of this notion, the EF1-tTA also mediated a persistent expression of luciferase in non-pregnant, multiparous mice (not shown).

### Persistent expression of hyperactive STAT5a under the control of the EF1-tTA delays postlactational remodeling

**Figure 6:**
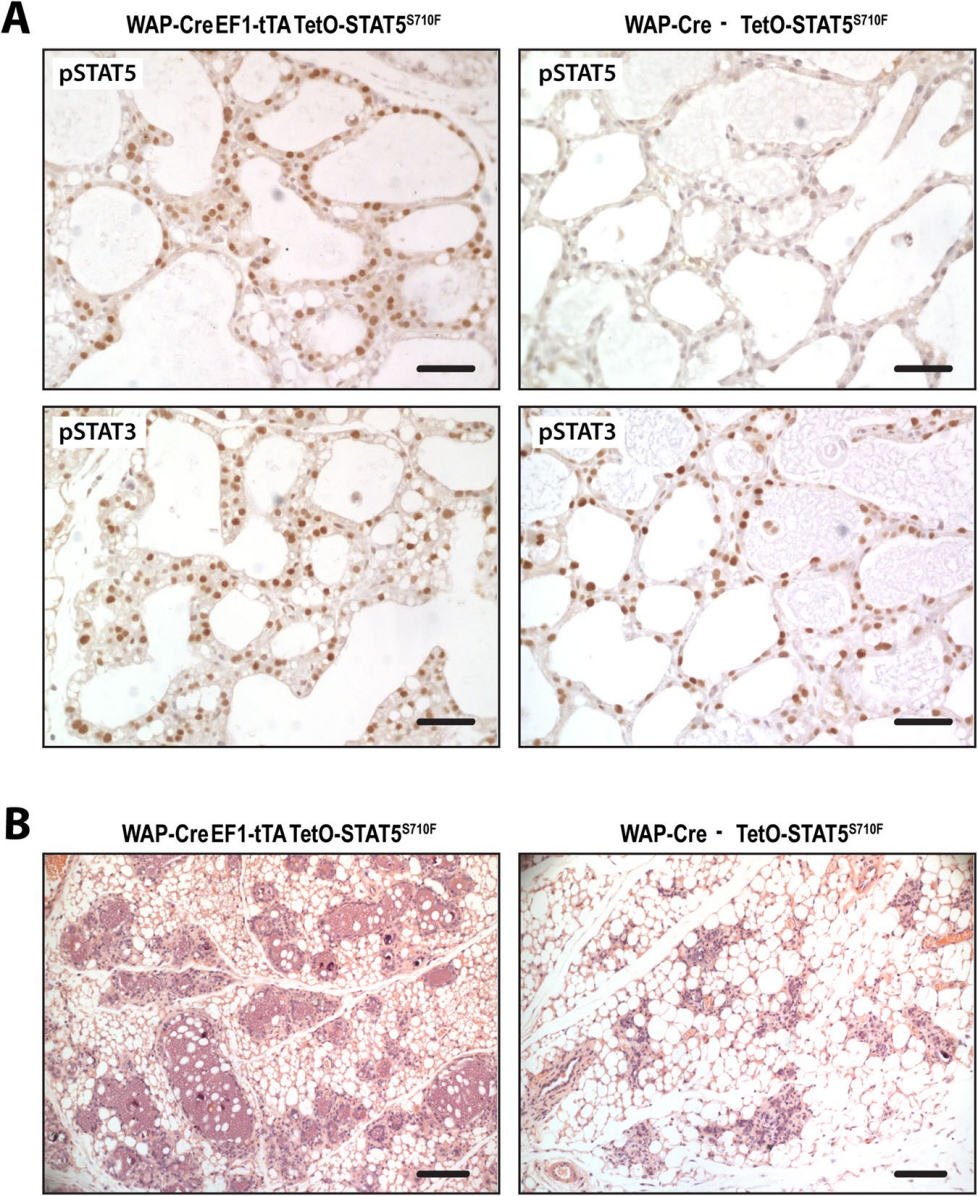
Sustained EF1-tTA-mediated expression of a hyperactive mutant of STAT5a (STAT5a^S710F^) delays postlactational remodeling despite timely activation of STAT3. Immunohistochemical staining of tyrosine-phosphorylated (i.e., active) STAT5 and STAT3 on histological sections of mammary gland tissues of a WAP-Cre EF1-LSL-tTA TetO-STAT5a^S710F^ triple transgenic mouse and a control female without the EF1-LSL-tTA transgene on the third day following the weaning of the young; bars, 50 µm. (**B**) Hematoxylin/Eosin-stained sections of a triple transgenic experimental female and control on day 5 of involution; bars, 100 µm.

The Signal Transducer and Activator of Transcription 5a (STAT5a) is an essential downstream mediator of prolactin signaling that promotes the differentiation and survival of functionally differentiated alveolar cells and the expression of milk protein genes, like *Wap*^18^. Within hours after weaning of the offspring, STAT5 is rapidly being dephosphorylated and remains largely in an inactive state during postlactational remodeling. At the same time, mammary epithelial cells show strong activation of STAT3 in response to IL-6 class inflammatory cytokines. The simultaneous switch in the activation of these STATs is essential for the timely remodeling of the gland to prevent severe inflammation (mastitis) and malignant transformation. We tested the functionality of the EF1-LSL-tTA in an experimental setting where we expressed a hyperactive mutant of STAT5a (TetO-STAT5a^S710F^) in functionally differentiated alveolar cells (WAP-Cre) and examined the biological consequence of a persistent activation of STAT5 during postlactational remodeling (Fig. 6A). While tyrosine-phosphorylated STAT5 was absent in the mammary glands of control females that did not carry the EF1-LSL-tTA knockin, WAP-Cre EF1-LSL-tTA TetO-STAT5a^S710F^ triple transgenics exhibited a sustained nuclear expression of active STAT5 in alveolar cells during postlactational involution. The EF1-tTA-mediated expression of active STAT5 did not cause any delay in the phosphorylation of STAT3, but it significantly decelerated the postlactational remodeling of the lobuloalveolar compartment (Fig. 6B). This observation suggested that the sustained expression of hyperactive STAT5 is sufficient to partially overwrite the proapoptotic role of active STAT3.

### Conditional Cre-mediated activation of the EF1-tTA

**Figure 7:**
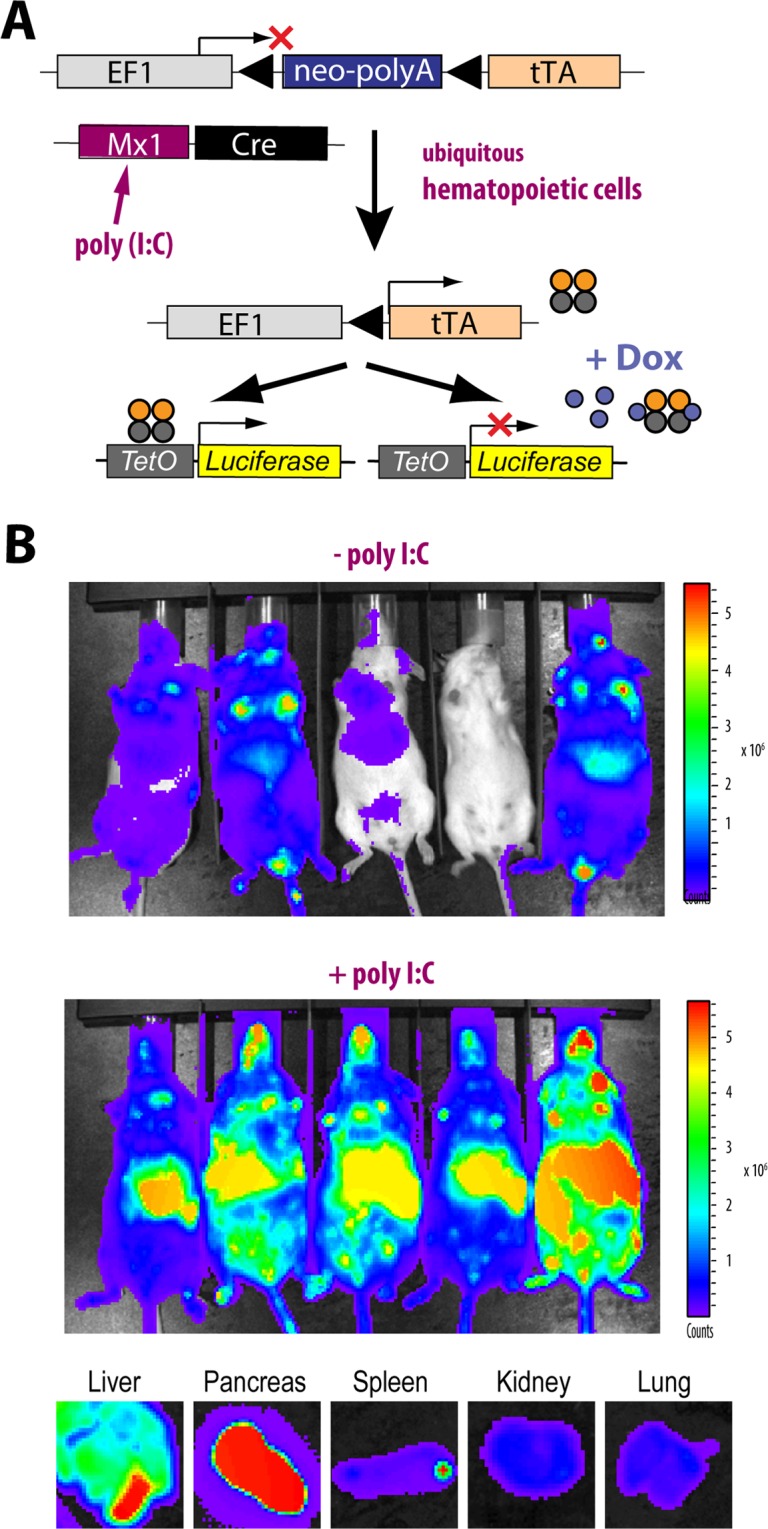
Ligand-inducible and Cre-mediated activation of the EF1-tTA and subsequent constitutive expression of TetO-driven responder genes. Schematic of the poly(I:C)-induced activation of the Mx1-Cre transgene that subsequently initiates a constitutive activation of the EF1-LSL-tTA and its responder genes in hematopoietic cells and other tissues. (**B**) *In vivo* bioluminescence imaging of Mx1-Cre EF1-LSL-tTA TetO-Luc triple transgenic mice prior to and 14 days after the last injection of poly(I:C). The lower panel shows bioluminescent images of selected organs from a poly(I:C)-treated triple transgenic mouse during necropsy.

The studies conducted with the WAP-Cre transgenics exemplify the usefulness of the EF1-LSL-tTA strain for a targeted overexpression of genes in particular cell types at defined developmental stages such as alveolar cells in lactating and involuting mammary glands. In addition to the Dox-mediated regulation of the EF1-tTA-driven responder genes, the timing of the initial activation of the EF1-tTA can be further refined through a ligand-controlled Cre recombinase. This might be a suitable experimental approach when it is the intent to overexpress a transgene long after a cell type has passed a particular differentiation stage or when a differentiated tissue undergoes extensive remodeling in response to an environmental cue (e.g., drug-induced pancreatitis). This strategy can also be used to activate a transgene at the same time in a given cell where another gene is being conditionally deleted (e.g., a study of cooperating functions of an oncogene with a tumor suppressor). In principle, the conditional Cre-mediated activation of the EF1-tTA can be achieved in two ways: 1) by stimulating the activity of an inducible promoter to express Cre (e.g., Mx1-Cre) or, 2) by using a Cre recombinase that is fused to a mutated ligand-binding domain of a nuclear hormone receptor like the estrogen receptor that can be activated with tamoxifen (Cre^ERT^). In a proof-of-principle experiment, we used the Mx1-Cre strain^19^ that expresses the recombinase under the control of the promoter of the interferon-inducible myxovirus resistance-1 (*Mx1*) gene to test whether the EF1-LSL-tTA can be activated in response to the administration of poly(I:C) [poly(I):poly(C)] (Fig. 7A). Despite a known activation of this transgene in diverse tissues, the Mx1-Cre has been most frequently used in studies of normal and malignant hematopoiesis. The bioluminescence imaging of Mx1-Cre EF1-LSL-tTA TetO-Luciferase triple transgenic mice showed that the Mx1-Cre was not silent in all animals prior to poly(I:C) injection, which is a known fact that has been reported by several research teams^20^. Nonetheless, there was a substantial increase in luciferase expression soon after the double-stranded polyribonucleotide was administered (Fig. 7B). We performed a necropsy of poly(I:C)-treated animals immediately following bioimaging and reexamined the bioluminescent signal within individual organs (Fig. 7B, lower panel). The strongest EF-tTA-mediated expression of luciferase was detected in the pancreas and liver, but the reporter was also uniformly active in cells of the spleen, kidney, and lung. Similar to the mammary gland, this was a noticeable improvement over the CAG-LSL-tTA strain, which was unsuitable for a transactivator-mediated overexpression of genes in hematopoietic cells^13^.

## Discussion

We have generated a new genetically engineered mouse strain that expresses the tet-controlled transactivator under the regulation of the ubiquitously active *Eef1a1* locus and in a Cre-mediated manner. This knockin mouse line is a universal tool that can be applied to express target genes in embryonic stem cells and in differentiated cell lineages. In contrast to the CAG-tTA and Rosa26-rtTA knockin lines that we tested, the EF1-tTA was able to reliably transactivate a high expression of transgenes in epithelial cells of secretory tissues such as mammary and salivary glands, as well as seminal vesicle (data not shown). Our previous experience was that the unmodified *Rosa26* locus induces only a moderate or low expression in a variety of tissues^13^. This notion was validated in a controlled study by Tchorz *et al*.^21^ where the authors employed a recombinase-mediated cassette exchange (RMCE) to replace the endogenous *Rosa26* promoter with commonly used regulatory elements that are ubiquitously active. Interestingly, the EF1 promoter exhibited the highest activation compared to the human cytomegalovirus (CMV) or CMV-enhanced chicken beta-actin (CAG) promoters as determined by flow cytometry and expression of GFP. Regarding the use of exogenous promoters for transgene construction, it might be important to note that not all commercially available EF1 promoters appear to be functionally identical^22^. As demonstrated in this study, a strong expression of a transgene can be achieved by a knockin into the endogenous *Eef1a1* locus without the need for introducing additional enhancer elements. An exceptionally high targeting frequency was accomplished in isogenic ES cells with the help of a promoter-less neomycin selection marker, which eliminates the need to maintain and screen an extended number of ES cell clones.

The functionality of the mammary-specific activation of the EF1-tTA was tested in two biological experiments: (1) the role of active STAT5 as a survival factor for secretory alveolar cells and its ability to delay the postlactational remodeling of the gland, and (2) the induction of multifocal mammary tumors in response to the expression of mutant KRAS. The persistent activation of the tTA under the constitutively active *Eef1a1* locus might be particularly well suited to study the onset and progression of cancer in mice. In contrast to lines that express the transactivator directly under a tissue-specific promoter (e.g., WAP, Pdx1), the oncogene is now under the control of the ubiquitously active *Eef1a1* locus, whose expression does not depend on the differentiation state of a transformed cell. In cases where the tTA/rtTA is linked to a tissue-specific promoter, the expression of an oncogene that is essential for tumor maintenance might be tethered to a certain differentiation state (e.g., luminal-type mammary cancers). Under such a scenario, a tumor cell that undergoes de- or trans-differentiation might lose the expression of the transactivator and therefore its essential oncogenic driver. Consequently, the loss of the oncogene will lead to a negative selection of this cell. This means that, under conditions where the driver oncogene is tethered to a particular differentiation state, the neoplastic cells may not readily assume biological features that drive cancer progression and metastatic dissemination like epithelial-mesenchymal transition (EMT) or contribute to a higher degree of tumor heterogeneity. Hence, the true transforming ability of an oncogene might be concealed because of this experimental shortcoming. The EF1-LSL-tTA strain could be useful to overcome these limitations and be employed in studies that (1) examine the biological significance of the same oncogenic driver in diverse cellular subtypes of an organ, (2) investigate their transdifferentiation and metastatic potential, and (3) determine whether heterogeneous cancer cell populations within a tumor still depend on the driver oncogene or whether they have become self-sufficient from a tumor-initiating oncogene as a consequence of de- or transdifferentiation. In cases where primary or metastatic cancers regress and residual tumor cells remain dormant following a Dox-induced suppression of the oncogene, it can be expected that the constitutively active EF1-tTA will effectively reactivate an oncogene in virtually all quiescent tumor cells following Dox withdrawal. This reawakening of tumor cells may lead to cancer recurrence or even cell death depending on the differentiation state of a dormant cancer cell^23–25^.

## Methods

### Construction of the EF1-LSL-tTA targeting vector

The pEF1 targeting vector, which contains the genomic sequence of the *Eef1a1* (EF1, EF1α) gene, was described earlier^26^. The 0.8 kb and 3.6 kb homology arms are separated by a multi-cloning site (MCS) containing restriction sites for *Pac*1, *Pme*1, and *Asc*1 (Fig. 1A). The *Pac*1 and *Asc*1 sites are being used for the directional insertion of the pSA-LoxP-pA cassette that contains a splice acceptor (SA) followed by a promoter-less neomycin (Neo) selection marker. Along with three polyA signals, the Neo serves as a transcriptional *Stop* sequence. The strategic location of two *lox*P sites upstream of the Neo and downstream of the triple polyA signal facilitates a Cre recombinase-mediated excision of the transcriptional *Stop* sequence. The coding sequence of the tetracycline controlled transactivator (tTA) was isolated as an *Eco*RI/*Bam*HI (blunt) fragment from the CMV-tTA plasmid (kindly provided by Lothar Hennighausen, NIH) and inserted into the multi-cloning site (*Eco*RI/*Not*I blunt) of the pSA-LoxP-pA cassette. The 5′ polyA site from the bovine growth hormone (bGHpA) mediates the termination of transcription of the tTA from the endogenous *Eef1a1* locus when the floxed Neo-polyA was excised by Cre recombinase. The final EF1-loxP-Stop-loxP-tTA (EF1-LSL-tTA) was constructed by cloning the pSA-LoxP-tTA-pA cassette into the pEF1 as shown in Fig. 1A.

### Gene targeting in ES cells, southern blot analysis, and PCR genotyping

The EF1-LSL-tTA targeting vector was linearized with *Asp*718, phenol/chloroform purified and electroporated into S1B6A embryonic stem (ES) cells, which were derived from an 129 Sv × C57BL/6 F1 embryo. The selection of targeted ES cells with G418 and expansion of individual ES cell clones were performed as described by Piovan *et al*.^27^ at the Genetically Engineered Mouse Modeling Core (GEMMC) at The Ohio State University. To validate the correct insertion of the LSL-tTA into the EF1 gene, we performed a Southern blot analysis. Genomic DNA from ES cell clones (15 μg) was digested overnight with *Eco*RV at 37 °C and separated on a 0.7% agarose gel. The DNA was denatured and transferred onto a nylon membrane (Genescreen Plus, Perkin Elmer). The 5′ external probe was 616 bp in size and isolated by *Xba*I/*Sac*II restriction digest as described previously^26^. The probe was labeled with ^32^P using the Random Primed DNA Labeling kit (Roche), and the membranes were hybridized with the probe at 65 °C overnight. On the next day, the membranes were washed in 0.1x SSC buffer containing SDS and exposed for 48 hours to a KODAK XOMAT-AR film at −80 °C. The *Eco*RV Southern analysis yielded two distinct bands of 5.5 kb for the *wildtype* and 3.85 kb in size for the targeted EF1-LSL-tTA knockin allele. Following the transmission of the targeted allele through the germline of male chimeric mice as validated by Southern blot, we used PCR genotyping on founder mice and their offspring. The location of primer sets 2452/2131 (5′- CCG CAA TAG TCA CCT CGG GCT T -3′ and 5′- TTC GGA GCA CAT GTC CGA CG -3′) and 2453/2454 (5′- CGA CTG TGC CTT CTA GTT GCC -3′ and 5′- AAG AAT GAC TTC CAG CGC CAG GC -3′) within the EF1-LSL-tTA sequence are illustrated in Fig. 1B. These two primer sets yielded PCR products of 423 bp and 315 bp in size regardless of whether an animal carries an additional Cre recombinase transgene or not (Fig. 1C). The combinatorial use of primers 2452 and 2454 will produce a 160 bp PCR product for the *Eef1a1* wildtype allele, and primers 2452 and 599 (5′- GCC AAT ACA GTG TAG GCT GC-3′) can be used to detect a fragment of 470 bp in size that represents the activated EF1-tTA allele in cells that express Cre recombinase (Fig. 1B,C). The targeted allele was registered at the Mouse Genome Informatics (MGI) Database as *Eef1a1*^*tm1(tTA)Kuw*^ (MGI:6360534). Further information and requests for the EF1-LSL-tTA knockin mouse models should be directed to the Lead Contact, Kay-Uwe Wagner (wagnerk@karmanos.org).

### Additional genetically modified mouse strains

To study the tissue-specific activation of the EF1-LSL-tTA knockin, we utilized the following four transgenic strains that express Cre recombinase: Pdx1-Cre [Tg(Pdx1-cre)6Tuv]^28^, Mx1-Cre [Tg(Mx1-cre)1Cgn]^19^, as well as our MMTV-Cre and WAP-Cre mice [Tg(MMTV-Cre)4Mam; Tg(Wap-cre)11738Mam]^29^. To validate the expression profile of the Pdx1-Cre transgene, we used the CAG-LSL-GFP reporter strain that was kindly provided by Dr. Miyazaki (Osaka University)^30^. The TetO-H2B-GFP (Tg(tetO-HIST1H2BJ/GFP)47Efu/J) and TetO-Luciferase reporter lines have been described earlier^8,31^. For biological experiments assessing the gain-of-function of mutant KRAS and hyperactive STAT5 in the mammary gland, we used TetO-Kras^G12D^ transgenics [Tg(tetO-Kras2)12Hev/J]^32^ and the TetO-STAT5a^S710F^ strain generated by our team [Tg(tetO-Stat5a*S710F,-luc)11676Kuw]^11^. This study was conducted in accordance with the recommendations in the Guide for the Care and Use of Laboratory Animals of the National Institutes of Health. The animal study protocols were approved by the Institutional Animal Care and Use Committee of the Nebraska Medical Center and Wayne State University.

### *In vivo* bioluminescence imaging, doxycycline administration, and poly(I:C) treatment

The administration of doxycycline (Dox) as medicated drinking water and the use of the IVIS200 (Caliper Life Sciences, Alameda, CA) for *in vivo* bioluminescence imaging were described previously^8,13,33^. Mx1-Cre transgenic mice were injected five times with 250 μg poly(I:C) [poly(I):poly(C)] over a period of 10 days. Bioluminescence imaging was performed prior to and 14 days after the last injection of poly(I:C).

### Histologic analysis and immunostaining

Fresh tissue samples from various organs including whole mounts of inguinal mammary glands were prepared by spreading them on glass slides. These unfixed specimens were examined with a Discovery.V8 fluorescence stereoscope (Carl Zeiss, Inc.) for GFP expression or the IVIS200 bioluminescence imager to assess luciferase activity. Mammary gland whole mounts were fixed for 16 hours in Carnoy’s solution, rehydrated, stained with Carmine Alum overnight, dehydrated, and mounted. For histological examination, tissues were fixed overnight in 10% buffered formalin (Fisher Scientific Company) and stored in 70% ethanol prior to paraffin embedding. Sections of 5 µm were deparaffinized three times in 100% Histo-Clear (National Diagnostics), rehydrated in decreasing concentrations of ethanol, and washed for 5 min in 1x PBS. Slides were stained with Hematoxylin and Eosin (H&E) for routine histology. A basic protocol for immunohistochemistry or immunofluorescent staining on paraffin-embedded specimens was described elsewhere^8^. We used the following primary antibodies: anti-GFP (GFP-1020) from Aves Labs; α-CK14 (PRB-155P) from Covance; α-CK8 (TROMA-I) from the Developmental Studies Hybridoma Bank; α-anti-pStat3 (pTyr705) from Cell Signaling; and α-pStat5A/B (pTyr694/699) from Upstate Biotechnology. For visualization of the specific targets, we used corresponding biotinylated secondary antibodies and Vectastain Elite ABC kits (Vector Laboratories), or secondary antibodies conjugated to Alexa Fluor dyes 488 and 594 (Invitrogen). Stained slides were examined with an Axio Imager microscope (Carl Zeiss, Inc.) equipped with a SPOT FLEX camera (Diagnostic Instruments, Inc.).
